# Real-World Experience with Carbidopa-Levodopa Extended-Release Capsules (Rytary^®^): Results of a Nationwide Dose Conversion Survey

**DOI:** 10.1155/2021/6638088

**Published:** 2021-02-19

**Authors:** Robert A. Hauser, Ghazal Banisadr, Kara Vuong, David Freilich, Stanley Fisher, Richard D'Souza

**Affiliations:** ^1^University of South Florida, Tampa, FL, USA; ^2^Amneal Pharmaceuticals, Bridgewater, NJ, USA

## Abstract

**Background:**

The introduction of carbidopa-levodopa extended-release (CD-LD ER) capsules (Rytary®) did not go as smoothly as expected, largely due to difficulty around dose conversion from available immediate-release (IR) levodopa (LD) formulations. The dose conversion table in the CD-LD ER prescribing information was similar to the table used in the pivotal clinical trial and is considered by many prescribing HCPs to be less than optimal. By the end of the dose conversion period in that trial, dosing in 76% of subjects was adjusted for symptom control; roughly 60% of patients required a higher dose and about half required more frequent administration than the recommended TID dosing.

**Objective:**

The primary objective of our nationwide (US) survey was to determine the dose conversion strategy most commonly employed by CD-LD ER frequent prescribers. The survey also aimed to explore additional features regarding CD-LD ER use in clinical practice.

**Methods:**

A survey consisting of 21 multiple-choice questions was developed and administered to experts in the use of CD-LD ER, based on prescription volume.

**Results:**

Of the 394 HCPs who were invited to participate, 90 (23%) HCPs completed the survey. All respondents were aware of the dose conversion table; the largest group did not find the table to be helpful and did not use it to convert patients to CD-LD ER. The most common strategy in calculating the CD-LD ER dose was based on the total daily LD IR dose, with the majority of that group initiating dose conversion by doubling the total daily LD dose from CD-LD IR and administering CD-LD ER one less time per day.

**Conclusion:**

Overall, most survey respondents agreed that a good starting point for CD-LD ER conversion could be doubling the daily LD IR dose and administering it one time less frequently. Moreover, rapid patient follow-up after initial dose conversion to allow for further dose adjustments plays a critical role in achieving success. Gaining experience over time is important for satisfactory conversion.

## 1. Introduction

It has been five years since carbidopa-levodopa extended-release (CD-LD ER) capsules (Rytary®) were introduced in the US, addressing the critical need for an oral long-acting LD to mitigate motor fluctuations in patients treated with short-acting preparations. Real-world experience can now serve as a guide for best practice utilization of CD-LD ER, including successful conversion from CD-LD immediate-release (IR).

CD-LD ER is a novel, multi-particulate formulation of LD and CD in a 4 : 1 ratio that contains both IR and ER components, as well as an absorption-modifying agent. This distinct formulation results in a pharmacokinetic profile that is different from CD-LD IR [1]. After a single dose of CD-LD ER, LD plasma concentrations rise quickly and remain above 50% C_max_ for approximately 4-5 hours [2, 3]. In contrast, after a single dose of CD-LD IR, LD plasma concentrations rise quickly but soon fall, remaining above 50% C_max_ for only about 1.5 hours [2, 3].

Converting patients from existing CD-LD formulations to CD-LD ER requires specialized knowledge. Dosages of CD-LD ER are not interchangeable with other CD and LD products on a 1 : 1 basis [RYTARY PI]. Based on pharmacokinetics, if one wishes to match the LD C_max_ associated with a fixed dose of CD-LD IR it would require approximately three times the LD in a single CD-LD ER dose [4].

The dosing table in the CD-LD ER prescribing information provides guidance for converting a patient's daily dosing regimen from CD-LD IR to CD-LD ER (RYTARY PI). This dosing table is the one that was provided to investigators for use in the pivotal trial of CD-LD ER (ADVANCE-PD) [5]. This conversion table was created by developers of CD-LD ER based on its pharmacokinetics and anticipated clinical effects [3]. In the pivotal trial, investigators were to use this dose conversion table for patient's initial conversion from CD-LD IR to CD-LD ER TID and then dose adjustments were permitted every few days during a 6-week open-label dose conversion phase of the study. Post hoc analysis of dose conversion in the ADVANCE-PD study found that 59.5% of patients who were successfully converted ended up on a higher daily LD dose than recommended in the conversion table and 15.5% ended up on a lower dose. The total daily LD dose from CD-LD ER at the end of the conversion period was 1.96 times the total daily LD dose from CD-LD IR at the beginning of the conversion period. In addition, 52.2% of patients were taking CD-LD ER 3x/day, 39.7% were taking it 4x/day, and 7.9% were taking it 5x/day [6]. It should also be noted that, at the end of the study, patients treated with CD-LD ER were still experiencing a mean of 3.87 hours daily OFF time, suggesting that further increases in dosing frequency might have been beneficial [5].

To better understand how experts use CD-LD ER in clinical practice, in 2019, Amneal Pharmaceuticals sponsored an open-ended phone survey of 10 highly experienced movement disorder specialists (unpublished). This small survey indicated that strategies being used to convert from CD-LD IR to CD-LD ER were variable, but also suggested a common strategy of converting from CD-LD IR to CD-LD ER at twice the total daily LD dose of CD-LD IR and administering it one time less frequently each day. This strategy was discussed at a CD-LD ER consensus advisory board and published by Espay et al. in 2017 [7]. To expand on these preliminary findings and learn more about CD-LD ER expert use, we conducted a national survey among US-based general neurologists and movement disorder specialists (MDS) to obtain information regarding real-world best practices on the use of CD-LD ER, including dose conversion.

## 2. Methods

### 2.1. Survey Design

A survey consisting of 21 multiple-choice questions was developed to obtain respondent data in five areas:

1. Respondent practice type and depth of experience prescribing CD-LD (Q1–5, 7)
2. Depth of experience in using CD-LD ER (Q6, 8, 9)
3. Dose conversion strategies for CD-LD ER (Q10–16)
4. Ideal patients for CD-LD ER treatment (Q17–20)
5. Reasons for discontinuing CD-LD ER (Q21)

### 2.2. Survey Participants

IQVIA, a healthcare data analytics company, utilized a 3rd party national claims database of approximately 9,800 healthcare providers (HCPs) to generate a respondent list. HCPs within the US, including Puerto Rico, who had eight CD-LD ER prescription transactions (TRx) per month over a prior one-year time period were considered eligible for participation. Based on expert consultation, a minimum of eight TRx per month was selected as a cutoff to define experienced CD-LD ER prescribers. From January 2020 through February 2020, eligible HCPs were invited in groups from the most experienced to the least experienced; as many groups as needed were generated until 100 responses were received, or the list was exhausted. HCPs were invited to participate via an e-mail that included instructions and a web link. HCPs for whom an e-mail could not be obtained were not invited to participate. If no response was received after two to three reminder emails, HCPs were then contacted via a phone call. Respondents were reimbursed for participation.

To ensure respondent anonymity, ClarityCo, a strategic insight gathering vendor, recruited respondents, hosted the survey online, disbursed respondent reimbursement, and collected responses. ClarityCo was chosen as the host vendor based on experience and prior collaboration. The survey was conducted in compliance with the Health Information Portability and Accountability Act (HIPAA) and related guidelines set forth by the Council of American Survey Research Organizations (CASRO).

### 2.3. Data Collection, Analysis, and Statistics

The primary objective of the survey was to determine if experienced CD-LD ER prescribers utilized a similar dose conversion strategy to those discovered during the small phone survey mentioned above. Once a respondent finished the online survey, his or her responses were collected and aggregated by ClarityCo. Blinded results were then tabulated using Excel for all eligible respondents who completed the survey. For survey questions 17–21, respondents were given a question with a number of possible factors to consider (a, b, c, d, etc.) to weigh in their response. For each subquestion, respondents provided an ordinal rated response on a scale of 0–10 to evaluate under what conditions they would convert to or utilize CD-LD ER. For data representation, the number space was re-mapped to a five-point (1–5) scale. Responses were grouped into the following five categories: very unlikely/very unimportant (rating of 0 or1), unlikely/unimportant (2 or 3), ambivalent (4, 5, or 6), likely/important (7 or 8), and very likely/very important (9 or 10).

A subanalysis of responses based on respondent answers to survey questions 6, 8, and 9 was done. Questions 6 and 8 queried the number of CD-LD ER prescriptions and conversion attempts in the prior year, respectively. Respondent choices for both questions were <10, 11–50, 51–100, and >100. Question 9 asked what percentage of patients was successfully converted to CD-LD ER in the prior year: ≤20%, 21–40%, 41–60%, 61–80%, and 81–100%. Success was defined as “remained on CD-LD ER for at least 3 months.”

In order to examine if dosing strategies differed in the very top tier of CD-LD ER users, results were analyzed for those respondents who reported >51 CD-LD ER patient conversion attempts (frequent converters), and those who reported ≥61% successful conversion (successful converters).

## 3. Results

Of the 394 HCPs who were invited to participate, 90 HCPs (23%) completed the survey. While the initial enrollment target was 100 responses, after 90 responses recruitment dramatically slowed and the survey was closed shortly thereafter. A total of 4 groups of HCPs were needed and asked to fill out the survey in order to obtain these 90 replies. The first group of HCPs had the highest survey response rate at 38% (*n* = 38/100). The second, third, and fourth group of HCPs had response rates of 22% (*n* = 22/100), 16% (*n* = 24/152), and 15% (*n* = 6/42), respectively.

Respondent practice experience, type of practice, and quantitative information regarding PD experience are provided in Table 1 (survey questions 1–8). Most respondents who saw greater than 51 PD patients per month (89%) were MDS, primarily treating PD patients (91%) and practiced in either an academic institution (53%) or community-based setting with academic affiliation (24%). Overall, respondents reported writing a high number of CD-LD IR prescriptions per month (question 5) and a lower number of CD-LD ER prescriptions (question 6). The majority of respondents reported writing greater than 51 prescriptions per month for CD-LD IR (74%) and 11–50 CD-LD ER prescriptions per month (69%). Twenty-two percent (*n* = 20) of respondents reported writing greater than 51 CD-LD ER prescriptions per month.

### 3.1. Experience Converting to CD-LD ER (Survey Questions 6–16)

**Figure 1:**
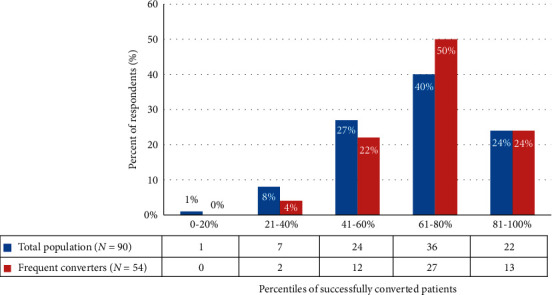
Respondents success rate of CD-LD IR to CD-LD ER conversion.

Question 8 asked respondents how many patients they attempted to convert to CD-LD ER in the previous year. Twenty-two percent selected more than 100 patients, 38% selected 51–100 patients, 38% selected 11–50 patients, and 2% selected less than 10 patients. Additionally, respondents were asked to gauge their success in converting patients to CD-LD ER in the prior year (survey question 9). Success was defined as patient “remained on CD-LD ER for at least three months.” Twenty-four percent of respondents reported a conversion success rate of 81–100%, 40% of respondents reported a conversion success rate of 61–80%, and 27% of respondents reported a conversion success rate of 41–60% (Figure 1: survey question 9).

All respondents were aware of the dosing table in the CD-LD ER prescribing information; however, 38% (*n* = 34/90) did not find the table to be helpful and did not use it to convert patients to CD-LD ER, 52% of respondents (*n* = 47/90) used the table along with their own calculations, and 10% (*n* = 9/90) found the dosing table helpful and used the table when converting patients (Table 2: survey question 10).

When asked how the CD-LD IR dose was used to convert to CD-LD ER, 49% (*n* = 44/90) of respondents reported they calculated the CD-LD ER dose based on the “*total daily* CD-LD IR dose”; 24% (*n* = 22/90) reported they based the CD-LD ER dose on an “other” strategy; and 20% (*n* = 18/90) reported they based it on the “*individual* CD-LD IR dose” (Table 2: survey question 11). Notably, only 7% reported using the dose conversion table from the prescribing information to convert patients.

Respondents who reported calculating the CD-LD ER dose based on either the “total daily CD-LD IR dose” (*n* = 44) or “individual CD-LD IR dose” (*n* = 18) were further queried on their dose conversion ratio in survey question 12 or 13, respectively. The majority of respondents who converted based on total daily CD-LD IR dose selected a dose conversion ratio of double (2*x*) the total daily CD-LD IR dose (80%; *n* = 35/44) (Table 3). Alternatively, respondents who converted based on the individual CD-LD IR dose most commonly reported a conversion ratio of 2.5–2.9x the individual CD-LD IR dose (50%; *n* = 9/18).

When considering dose conversion, frequency of dosing plays a critical role. In survey question 14, respondents were asked about their most commonly used dosing frequency strategy when converting patients from CD-LD IR to CD-LD ER. Approximately 1 in 4 respondents (27%) indicated utilizing an initial strategy of CD-LD IR frequency minus one to convert to CD-LD ER (Table 2). In addition, providers would commonly start with TID or QID dosing and adjust based on symptomatic control (34%). Overall, the majority of respondents (57%) favored a dose frequency reduction when converting from CD-LD IR to CD-LD ER.

**Figure 2:**
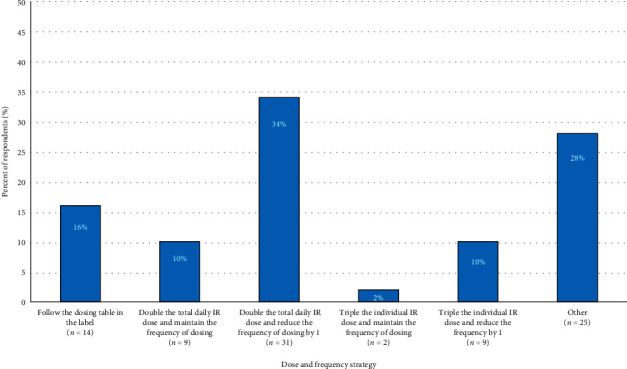
Recommended dosing and frequency strategy (*N* = 90).

To gain additional insight into expert guidance, we asked respondents “*If you were to teach a healthcare provider how to convert a patient from CD-LD IR to CD-LD ER, then which of the following strategies would you recommend?*” The most common response, chosen by 34% of respondents, was “double the total daily IR dose and reduce the frequency of dosing by one” (Figure 2).

During our previous phone survey, all participants strongly suggested rapid patient follow-up played a critical role in dose conversion success. Question 16 of the current survey asked “*How soon after initial conversion to CD-LD ER (RYTARY) do you or your staff seek feedback (phone calls or appointments) from the patient in order to determine if adjustments to the initial conversion schedule are required?*”; 74% of respondents reported following up within one week after CD-LD ER dose conversion.

### 3.2. *Appropriate Patients to Convert to CD-LD ER* (Survey Questions 17–21)

**Figure 3:**
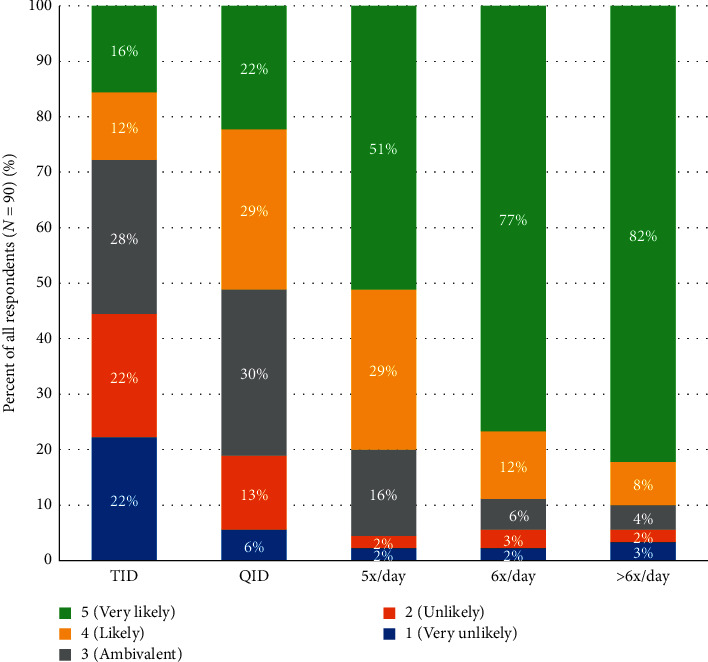
Likelihood of switching to CD-LD ER: multiple OFFs and no dyskinesia per CD-LD IR dosing frequency.

When asked about the most appropriate patients with multiple OFF episodes every day to convert from CD-LD IR to ER, respondents indicated that they were unlikely to convert patients on CD-LD IR TID dosing, but the percentage of respondents who indicated they would convert to CD-LD ER increased dramatically as the number of CD-LD IR doses increased (Figure 3).

**Figure 4:**
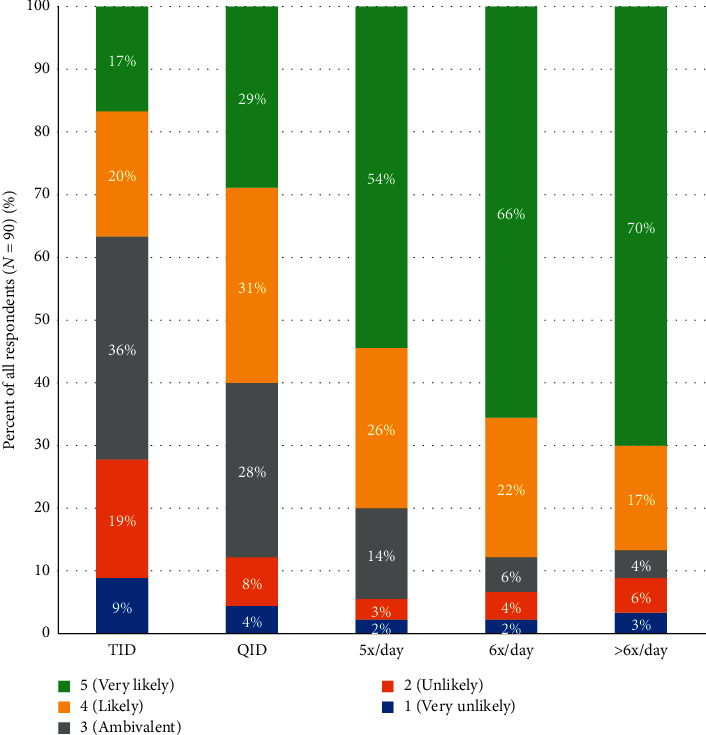
Likelihood of switching to CD-LD ER: multiple OFFs and dyskinesia per CD-LD IR dosing frequency.

Also, the percentage of respondents who indicated they would convert to CD-LD ER did not substantially change based on the presence of dyskinesia. For three times daily CD-LD IR dosing, 28% were very likely or likely to dose convert in patients with multiple OFF episodes and no dyskinesia, while 37% were very likely or likely to dose convert in patients with multiple OFF episodes and dyskinesia. For four times daily CD-LD IR dosing, 51% and 60%, respectively, were very likely or likely to dose convert. A strong transition occurred at 5x/day CD-LD IR. At this dosing interval, 80% of respondents were very likely or likely to convert patients with multiple OFF episodes with dyskinesia or without dyskinesia to CD-LD ER (Figure 3: survey question 17, Figure 4: survey question 18).

Question 19 asked respondents how likely they were to use CD-LD ER in three types of patients: newly diagnosed PD patients, early PD patients in need of LD treatment, and patients on LD treatment but with no OFF episodes. For each of these groups, respectively, 79%, 71%, and 79% of respondents indicated that they were unlikely or very unlikely to switch to CD-LD ER.

**Figure 5:**
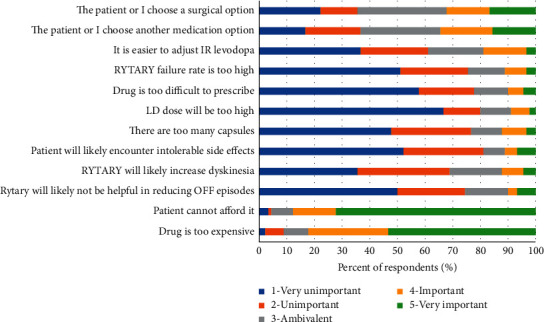
Importance rating of reasons why CD-LD ER would not be used in a patient with OFF episodes.

Figure 5 shows how respondents rated the importance of various factors when choosing not to use CD-LD ER in patients with OFF episodes (survey question 20). The primary reasons that were considered important or very important were affordability (87%) and cost (81%), while factors such as pill burden (23%), total LD dose (16%), side effects (23%), dyskinesia (27%), and inability to provide clinical benefit (24%) were not deemed concerns by a large percentage of respondents.

**Figure 6:**
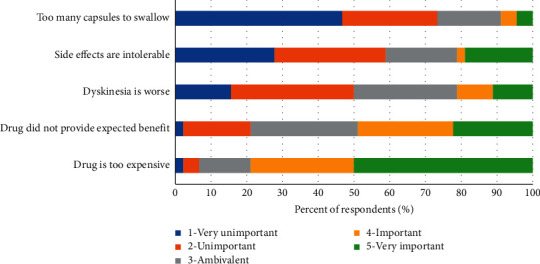
Importance rating of reasons why CD-LD ER is discontinued after initiation.

Figure 6 (survey question 21) illustrates respondents' impression of importance of various factors leading to patient discontinuation of CD-LD ER in their practice. Fifty percent rated “drug is too expensive” as very important and 29% rated this as important. Another reason for discontinuation was CD-LD ER not providing the expected benefit; 22% rated this as very important, 26% rated it as important, and 30% were ambivalent, making it the second most frequent reason for discontinuation after cost. Notably, side effects, too many capsules to swallow, and worsening dyskinesia were not considered important factors in patient discontinuations by a large percentage of respondents (24%–42%).

### 3.3. Subanalysis of Respondents Reporting Large Number of Patient Conversions or Success Rates

In the frequent converter group (*n* = 54), 74% of respondents (*n* = 40/54) reported successful conversion in 61–100% of patients. Forty-six percent of the respondents in this group indicated that the dosing table was not helpful and was not used in dose calculations. Fifty-nine percent of respondents in this group based the CD-LD ER conversion on the total daily CD-LD IR dose (survey question 11). This includes respondents who selected options b and d “other” but whose free response for “other” included a discussion of dose conversion based on the total daily LD IR dose.

In the successful converter group (*n* = 58), 50% (*n* = 29/58) of respondents indicated that the dosing table in the prescribing information was “NOT helpful, and I DO NOT use it to convert my patients.” When asked about conversion strategy, 57% (*n* = 33/58) of respondents selected “My strategy is most commonly based on the TOTAL DAILY LD dose of IR.” Additionally, almost all respondents who chose this option selected a dose ratio of twice the daily CD-LD IR dose (88%; *n* = 28/33).

## 4. Discussion

Although most clinicians experienced in treating PD patients are very adept at using CD-LD IR, understanding how to maximize the utility of CD-LD ER upon its initial introduction to the market proved to be challenging. The CD-LD ER prescribing information includes the dose conversion table that was created for the clinical development program, but this conversion regimen turned out to be less than optimal. Therefore, important insights can be gained from real-world clinical experience over the last 5 years. The current survey aimed to uncover patterns in how to best convert patients from CD-LD IR to CD-LD ER and identify where in the patient journey CD-LD ER conversion might be most appropriate, based on the collective experience of frequent prescribers.

Our results, reflecting the opinion of 90 frequent prescribers, amplified our previous findings of a small phone survey and showed that the majority of surveyed clinicians, when converting patients with motor fluctuations from CD-LD IR to CD-LD ER, use a conversion strategy based on the total daily CD-LD IR dose, with most of them choosing to double the total daily LD IR dose as the starting point. This strategy was initially proposed by Hauser [4] and subsequently endorsed by 11 MDS highly experienced with CD-LD ER in clinical trials and clinical practice [7]. Further, over half of survey respondents utilize a strategy of selecting a daily frequency of administration that is one less than the CD-LD IR daily frequency.

We found strong consensus that the dosing table in the CD-LD ER prescribing information offered little utility. This observation is reflected in the phase 3 trial where roughly 60% of patients required a dosage higher than those recommended by the dosing table for their initial regimens [5]. In addition, 47.6% of patients required an increase in dosing frequency from the initial TID dosing recommended in the label.

Many PD patients are initially treated with CD-LD IR TID. With disease progression and the emergence of increasing OFF time, CD-LD IR dosing frequency is typically increased. In our survey, the percentage of responders who indicated they would convert to CD-LD ER to manage motor fluctuations appeared to be directly related to the frequency of CD-LD IR doses, regardless of the presence or absence of dyskinesia, suggesting that dyskinesia is not an important factor in their decision to convert to CD-LD ER. CD-LD IR doses of five times per day was the pivot point at which there was a large jump in respondents who were likely or very likely to convert to CD-LD ER. Furthermore, the majority of respondents stated that they were unlikely or very unlikely to switch newly diagnosed PD patients, early PD patients, and patients on LD treatment but with no OFF episodes to CD-LD ER, indicating that motor fluctuations continue to be the driving force behind current use of CD-LD ER. In addition, insight into factors leading to discontinuations also provides real-world guidance for successful conversion.

Our study has several limitations. Utilizing a survey is dependent on respondent recollection and recall bias is inherent. Although the sample size seems limited, the study aimed at gathering insights from prescribers most experienced using CD-LD ER and we believe results reflect expert consensus. The survey did not capture methods of dose adjustment after initial dose conversion, nor did it examine details of the extent of patient follow-up.

Overall, this survey, based on 5 years of real-world clinical experience, confirmed findings from an earlier small phone survey (unpublished, 2019) and previous expert consensus statement [7]. Even though the initial dose selection for conversion from CD-LD IR to CD-LD ER varies by prescriber, most survey respondents agreed that doubling the LD IR dose and reducing the frequency by one could be a good starting point to optimize clinical benefit.

## Figures and Tables

**Table 1:** Respondent practice characteristics.

	*n* (%)
*Specialty*	
GN treating some PD pts	4 (4)
GN treating mostly PD pts	1 (1)
MDS treating some PD pts	3 (3)
MDS treating mostly PD pts	82 (91)

*Type of practice*	
Academic institution	48 (53)
Community-based facility	20 (22)
Community-based with academic affiliation	22 (24)

*Years in practice*	
<10	33 (37)
11–20	38 (42)
>20	19 (21)

*Average no. of PD pts seen/month*	
<10	0 (0)
11–50	10 (11)
51–100	39 (43)
>100	41 (46)

*CD-LD IR Rx/month*	
<10	3 (3)
11–50	20 (22)
51–100	45 (50)
>100	22 (24)

*CD-LD ER Rx/month*	
<10	8 (9)
11–50	62 (69)
51–100	14 (15)
>100	6 (7)

*GN* = general neurologist; MDS = movement disorder specialist; PD = Parkinson's disease; CD-LD IR = carbidopa-levodopa immediate-release; CD-LD ER = carbidopa-levodopa extended-release.

**Table 2:** Strategy for determining CD-LD ER dose and frequency (*N* = 90).

	*n* (%)
*Dosing table (survey question 10)*	
Not aware	0
Helpful to convert	9 (10)
Not helpful and do not use	34 (38)
Somewhat useful along with own calculations	47 (52)

*Dose conversion strategy (survey question 11)*	
Based on the label	6 (7)
Based on total daily CD-LD IR dose	44 (49)
Based on individual CD-LD IR dose	18 (20)
Other	22 (24)

*Dose frequency strategy (survey question 14)*	
TID and then adjust	20 (22)
QID and then adjust	11 (12)
CD-LD ER dose same as CD-LD IR dose	8 (9)
CD-LD ER dose one less than CD-LD IR dose	24 (27)
CD-LD ER TID or QID if CD-LD IR QID or 5x per day, respectively	27 (30)

CD-LD = carbidopa-levodopa; IR = immediate-release; ER = extended-release; TID = three times a day; QID = four times a day.

**Table 3:** CD-LD IR to CD-LD ER dose conversion ratio.

	*n* (%)
*Based on total daily CD-LD IR dose (n* *=* *44)*	
2x CD-LD IR dose	35 (80)
2.1–2.4x CD-LD IR dose	5 (11)
>2.4x CD-LD IR dose	1 (2)
Depends on CD-LD IR dose	3 (7)

*Based on individual CD-LD IR dose (n* *=* *18)*	
2.5–2.9x CD-LD IR dose	9 (50)
3x CD-LD IR dose	5 (28)
3.1–3.5x CD-LD IR dose	0
Depends on CD-LD IR dose	4 (22)

CD-LD = carbidopa-levodopa; IR = immediate-release; ER = extended-release.

## Data Availability

The data used to support the findings of this study are included within the article.

## Supplementary Materials

Supplementary Materials The survey questionnaire and conversion table from the CD-LD ER label are provided in supplementary materials (S1 and S2, respectively).
