# Supplementary material for: Real-World Experience with Carbidopa-Levodopa Extended-Release Capsules (Rytary®): Results of a Nationwide Dose Conversion Survey
Source: Parkinsons Dis. 2021 Feb 19;2021:6638088. doi: 10.1155/2021/6638088 (PMC7914099; doi:10.1155/2021/6638088)
Supplement: Supplementary Materials — The survey questionnaire and conversion table from the CD-LD ER label are provided in supplementary materials (S1 and S2, respectively). [file 6638088.f1.docx]

**Supplementary Material**

**S1. Survey Questionnaire**

1. **What best describes your specialty area?**
2. General neurologist treating some PD patients
3. General neurologist treating mostly PD patients
4. Movement disorder specialist treating some PD patients
5. Movement disorder specialist treating mostly PD patients
6. **What best describes your practice?**
7. Academic institution
8. Community-based facility
9. Community-based facility with an academic affiliation
10. **How many years have you been in practice?**
11. Less than 10
12. 11-20
13. More than 20
14. **How many Parkinson’s patients do you see in an average per month?**
15. Less than 10
16. 11-50
17. 51-100
18. More than 100
19. **How many prescriptions for immediate release (IR) carbidopa/levodopa (CD-LD) do you write per month?**
20. Less than 10
21. 11-50
22. 51-100
23. More than 100
24. **How many prescriptions for RYTARY (CD-LD extended-release capsules) do you write per month?**
25. Less than 10
26. 11-50
27. 51-100
28. More than 100
29. **How many prescriptions for CD-LD/entacapone do you write per month?**
30. Less than 10
31. 11-50
32. 51-100
33. More than 100
34. **Approximately how many patients in total have you attempted to convert from another levodopa formulation to RYTARY in the past year?**
35. Less than 10
36. 11-50
37. 51-100
38. More than 100
39. **Of the patients who you attempted to convert to RYTARY in the past year, approximately what percentage was successfully converted? (i.e., remained on RYTARY for at least 3 months).**
    1. 0-20%
    2. 21-40%
    3. 41-60%
    4. 61-80%
    5. 81-100%
40. **What has been your experience with the dosing table in the RYTARY label?**
41. I am not aware of the dosing table in the RYTARY label.
42. It is helpful and I use it to convert my patients.
43. It is NOT helpful and I DO NOT use it to convert my patients.
44. It is somewhat useful and I use it along with my own calculations.
45. **What is your most commonly used strategy to convert patients from carbidopa/levodopa IR to RYTARY?**
46. My strategy is most commonly based on the conversion table in the label.
47. My strategy is most commonly based on the TOTAL DAILY levodopa dose of IR (for example, RYTARY total daily levodopa milligram dose is approximately 2 times the original levodopa IR daily milligram dose).

**[GO TO QUESTION 12]**

1. My strategy is most commonly based on the INDIVUDUAL dose of levodopa IR used throughout the day (for example, individual RYTARY milligram doses are approximately 3 times the original individual levodopa IR milligram doses used throughout the day).

**[GO TO QUESTION 13]**

1. Other (please specify) _________________________

[GO TO QUESTION 14]

1. **What is your most commonly used conversion ratio based on the TOTAL DAILY dose of IR?**
   1. 2 X the total daily IR levodopa dose.
   2. 2.1 - 2.4 X the total daily IR levodopa dose.
   3. > 2.4 X the total daily IR levodopa dose.
   4. It depends on the total daily IR levodopa dose.
2. **What is your most commonly used conversion ratio based on the INDIVIDUAL dose of IR?**
   1. 2.5 - 2.9 X the individual IR levodopa dose.
   2. 3 X the individual IR levodopa dose.
   3. 3.1 - 3.5 X the individual IR dose.
   4. It depends on the individual IR levodopa dose.
3. **What is your most commonly used dose frequency strategy when converting patients from levodopa IR to RYTARY?**
   1. Start TID and then adjust from there.
   2. Start QID and then adjust from there.
   3. Give RYTARY as frequently as IR.
   4. Give RYTARY at a frequency of 1 LESS THAN how often IR was given.
   5. Give RYTARY TID if levodopa IR was given QID; give RYTARY QID if levodopa IR was given 5 times or more per day.
4. **If you were to teach a healthcare provider how to convert a patient from levodopa IR to RYTARY, then which of the following strategies would you recommend?**
5. Follow the dosing table in the label.
6. Double the total daily IR dose and maintain the frequency of dosing.
7. Double the total daily IR dose and reduce the frequency of dosing by 1.
8. Triple the individual IR dose and maintain the frequency of dosing.
9. Triple the individual IR dose and reduce the frequency by 1.
10. Other: ____________________________________
11. **How soon after initial conversion to RYTARY do you or your staff seek feedback (phone calls or appointments) from the patient in order to determine if adjustments to the initial conversion schedule are required?**
    1. 1 - 3 days
    2. 4 - 7 days
    3. 2 - 4 weeks
    4. 2 - 4 months
12. **For the following patients with multiple OFF episodes every day and NO dyskinesia, how likely are you to switch them to RYTARY?**

**(Rating scale: 0 not at all likely, 10 highly likely)**

1. Patient is on levodopa IR TID
2. Patient is on levodopa IR QID
3. Patient on levodopa IR 5 times per day
4. Patient is on levodopa IR 6 times per day
5. Patients on levodopa IR > 6 times per day
6. **For the following patients with multiple OFF episodes every day AND dyskinesia, how likely are you to switch them to RYTARY?**

**(Rating scale: 0-not at all likely, 10-highly likely)**

1. Patient is on levodopa IR TID
2. Patient is on levodopa IR QID
3. Patient on levodopa IR 5 times per day
4. Patient is on levodopa IR 6 times per day
5. Patients on levodopa IR > 6 times per day
6. **How likely are you to initiate RYTARY or switch from levodopa IR to RYTARY for these types of patients?**

**(Rating scale: 0-not at all likely, 10-highly likely)**

1. Newly diagnosed PD patient
2. In a patient with early PD, in need of levodopa therapy
3. In a patient on levodopa IR with no OFF episodes
4. **How important are each of the following when you elect NOT to use RYTARY in a patient with OFF episodes? (Rating scale: 0-not at all important, 10-very important)**
   1. Drug is too expensive.
   2. Patient cannot afford it.
   3. RYTARY will likely not be helpful in reducing OFF episodes.
   4. RYTARY will likely increase dyskinesia.
   5. Patient will likely encounter intolerable side effects.
   6. There are too many capsules.
   7. The levodopa dose will be too high.
   8. Drug is too difficult to prescribe.
   9. RYTARY failure rate is too high.
   10. It is easier to adjust IR levodopa.
   11. The patient or I choose another medication option.
   12. The patient or I choose a surgical option.
5. **How important are each of the following regarding how often RYTARY is discontinued after initiation in your practice (0-not at all important, 10-very important)**
6. Drug is too expensive.
7. Drug did not provide the expected benefit.
8. Dyskinesia is worse.
9. Side effects are intolerable.
10. Too many capsules to swallow.

**Additional Comments**

Please share any additional information that you deem useful based on your clinical perspective and experience with RYATRY and other CD-LD medications.

**S2. Conversion from Immediate-Release Carbidopa-Levodopa to RYTARY [RYTARY PI]**

| **Total Daily Dose of**  **Levodopa in**  **Immediate-Release**  **Carbidopa-**  **Levodopa** | **Recommended Starting Dosage of RYTARY** | |
| --- | --- | --- |
|  | **Total Daily Dose of Levodopa**  **In RYTARY** | **RYTARY Dosing Regimen** |
| 400 mg to 549 mg | 855 mg | 3 capsules RYTARY 23.75 mg / 95 mg taken TID^a^ |
| 550 mg to 749 mg | 1140 mg | 4 capsules RYTARY 23.75 mg / 95 mg taken TID |
| 750 mg to 949 mg | 1305 mg | 3 capsules RYTARY 36.25 mg / 145 mg taken TID |
| 950 mg to 1249 mg | 1755 mg | 3 capsules RYTARY 48.75 mg / 195 mg taken TID |
| Equal to or greater  than 1250 mg | 2340 mg or | 4 capsules RYTARY 48.75 mg / 195 mg taken TID  or |
|  | 2205 mg | 3 capsules RYTARY 61.25 mg / 245 mg taken TID |

^a^ TID: three times a day
